# Medical liability in paediatric robotic surgery – a current concept and a literature review

**DOI:** 10.1007/s11701-026-04033-w

**Published:** 2026-10-05

**Authors:** Ciro Esposito, Anna Gargiulo, Claudia Di Mento, Marco Castagnetti, Vincenzo Coppola, Francesco Tedesco, Roberta Guglielmini, Maria Luisa Pirone, Chiara Caporale, Maria Escolino

**Affiliations:** 1 Pediatric Surgery Unit, Department of Translational Medical Sciences, University of Naples Federico II, Naples, Italy; 2 Forensic Medicine Department, Naples 3 South Local Health Authority, Naples, Italy; 3 Pediatric Urology, Bambino Gesù Children Hospital, Rome, Italy

**Keywords:** Pediatric surgery, Robotic surgery, Complication, Medical liability, Equipment failure

## Abstract

Robotic-assisted surgery offers significant technical advantages in pediatric procedures but presents complex, unresolved medico-legal challenges regarding liability. We retrospectively reviewed our ten-year experience comprising 205 pediatric robotic procedures. Technical issues related to the robot were encountered in 7 cases (3.4%). These included joystick latency (3/7), coagulation/cutting pedal malfunctions (2/7), and accidental power cable disconnections causing system shutdowns (2/7). A literature review was conducted to assess the current international state of the art regarding robotic medical liability. Although the technical events at our center did not result in clinical complications, they highlighted significant potential risks. The literature shows that specific guidelines governing human, product, and algorithm-related liability are still lacking. Robotic surgery shifts the focus from an exclusively fault-based liability of the surgeon to a network-based system involving multiple stakeholders: the surgeon, the healthcare institution, and the manufacturer. Determining whether an adverse event stems from human error, algorithmic malfunction, or a combination of both remains a critical challenge. Unlike open or laparoscopic surgery where liability is primarily attributed to the operator, robotic surgery involves a complex interaction between the surgeon’s console inputs and the manufacturer’s technology. Consequently, from a medico-legal standpoint, responsibility for complications should be shared among the surgeon, the hospital, and the device manufacturer.

## Introduction

Robotic Assisted Surgery (RAS) represents an expanding frontier in pediatric surgery, offering numerous advantages for both surgeons and patients, including enhanced precision, tremor elimination, motion scaling, and high-definition 3D visualization [1, 2]. Unlike laparoscopic surgery, where the operator directly holds the instruments, RAS positions the surgeon at a console manipulating joysticks, while the robotic system handles and maneuvers the instruments at the operating Tables [1–4]. This novel human-machine integration—which merges robotics, artificial intelligence, and augmented reality—is poised to drive the evolution of surgical techniques toward semi-autonomous or fully autonomous pediatric procedures in the near future [3, 5–9]. However, this technological shift introduces technical, economic, ethical, and medico-legal challenges that require urgent clarification. In particular, the medico-legal implications of surgical complications remain highly controversial [10–12]. The fundamental issue in robotic medical liability is the current lack of specific guidelines governing not only human error but also product- and algorithm-related liability, especially as software and robotic systems increasingly influence the surgical act [8, 10, 11]. Indeed, robotic surgery shifts the paradigm from an exclusively fault-based liability of the physician to a network-based liability system involving multiple stakeholders: the surgeon, the healthcare institution, the manufacturer, and the algorithm [8]. Consequently, a critical challenge lies in determining whether an adverse event stems from human conduct, an algorithmic malfunction, or a combination of both [8]. It is therefore necessary to assess whether complications are attributable solely to the operator or to a confluence of factors involving the hospital—responsible for selecting and installing the technology—and the manufacturer, as multi-factorial technical malfunctions may be equally liable. Our group has previously investigated the medico-legal aspects of conventional laparoscopy [12, 13]. However, the use of robotic platforms redefines this issue, as the surgeon is positioned at a remote console while a robotic device manipulates the surgical instruments at the table. This paper clarifies these specific liability concepts within the context of pediatric surgery.

## Materials and methods

We retrospectively reviewed a 10-year experience in pediatric robotic surgery conducted by our department surgical team. The study period extends from November 2017 to August 2026. Patients were eligible for the robotic approach if they were older than 1 year and weighed more than 15 kg. Exclusion criteria included: weight < 15 kg, comorbidities such as hemodynamic instability, pulmonary hypoplasia, or other conditions contraindicating pneumoperitoneum according to anesthesiology evaluation, parental or patient refusal of the robotic approach.

All procedures included in the analysis were directly performed by the same two pediatric surgeons acting as first and second console surgeons. Interventions in which the surgeons participated solely as external mentors or consultants were excluded from the cohort. Robotic procedures were carried out at two centers: University Hospital Federico II, Naples, and Ruggi d’Aragona Hospital, Salerno.

All procedures were performed using the Da Vinci Xi Surgical System equipped with a double‑console configuration (Intuitive Surgical Inc., Sunnyvale, CA, USA). At our academic center— reflecting a model documented in 78.6% of Italian pediatric robotic surgery centers—the robotic platform is physically located within an operating suite managed by adult general surgery or adult urology departments, where it is shared among multiple surgical specialties. Consequently, the robotic platform’s setup and routine maintenance are managed by the adult surgical departments, while the pediatric team utilizes the shared operating suite exclusively for scheduled procedures. Within the overall cohort, we identified and analyzed all technical issues directly attributable to the robotic system. These included: (1) latency between joystick commands and robotic arm response, (2) malfunction of the cutting and coagulation pedals, (3) temporary system shutdowns caused by accidental disconnection of exposed power cables. In parallel, we conducted a narrative literature review aimed at contextualizing our institutional findings within the current international medico‑legal landscape. The review focused on Italian and European regulatory frameworks governing robotic surgery, product liability, institutional responsibility, and emerging algorithm‑related legal considerations. Sources were identified through targeted searches on PubMed and Google Scholar, including articles addressing liability in robotic surgery, organizational responsibility, and risk‑management models applicable to shared robotic platforms.

## Results

A total of 205 pediatric robotic procedures were analyzed. Robotic interventions were performed at two centers: University Hospital Federico II, Naples (195/205) and Ruggi d’Aragona Hospital, Salerno (10/205). The cohort consisted of 119 boys and 86 girls, with a median age of 9.5 years (range 2–17) and a median weight of 28.5 kg (range 17.5–83.5). All operative indications represented in the cohort included: ureteropelvic junction obstruction (62/205), vesicoureteral reflux (51/205), varicocele (22/205), ovarian tumors (10/205), primary obstructive megaureter (19/205), gallbladder disease (10/205), renal tumors (5/205), appendicitis (3/205), bilateral inguinal hernia (7/205), gastroesophageal reflux disease (7/205), renal stones (1/205), bladder stones (3/205), and urachal anomalies (5/205). Within the overall cohort, 7 technical incidents were recorded (3.4%). These consisted of latency between joystick commands and robotic arm response, (3/7) malfunction of the cutting and coagulation pedals (2/7), temporary system shutdowns caused by accidental disconnection of exposed power cables (2/7). Fortunately, none of these events resulted in perioperative or long-term complications for the patients. However, they carried a high potential for severe injuries (e.g. damaging major blood vessels, bowel loops, or parenchymal organs). In parallel, the international literature review revealed a significant regulatory void, as there are currently no specific guidelines governing product- and algorithm-related liability alongside human error, even though software and automated systems fundamentally influence the surgical act. Indeed, robotic surgery shifts the focus from an exclusively fault-based liability of the surgeon to a complex, network-based liability system involving multiple stakeholders: the surgeon, the healthcare institution, the device manufacturer, and the algorithmic software. Consequently, identifying whether an adverse event stems from human conduct, an algorithmic malfunction, or a combination of both represents a critical challenge. Furthermore, the analysis highlights that the hospital plays a substantial role in potential liability frameworks; since the platform is shared and physically located in an adult surgery operative room, the institution is directly responsible for selecting the technology to purchase, overseeing the installation process, and managing the safety of the installation site. Finally, the robot manufacturer bears strict liability for latent technical malfunctions that could compromise patient safety and lead to severe medico-legal litigation. The narrative literature review identified several recurring medico-legal themes relevant to robotic-assisted surgery in the pediatric setting. The literature specifically addressing pediatric robotic surgery remains limited, with the earliest publications focusing on the implications of robotic technology for surgical responsibility and the relationship between the surgeon and the robotic platform [8, 9, 14]. More recent publications have expanded the discussion to broader issues of institutional responsibility, medical-device liability, human–technology interaction, and emerging algorithmic and AI-related liability [19, 22, 23]. Five major themes emerged from the reviewed literature. First, robotic surgery introduces additional potential sources of technical and technology-mediated events, requiring differentiation between operator-related errors, robotic platform malfunction, and organizational or environmental factors. Second, responsibility may involve multiple stakeholders, including the surgeon, operating-room team, healthcare institution, and, depending on the specific circumstances and applicable legislation, the manufacturer or technology provider. Third, establishing causation represents a major medico-legal challenge because adverse events may result from the interaction of human, technical, and organizational factors. Fourth, institutional responsibility is particularly relevant in pediatric robotic surgery because robotic platforms are frequently shared between departments. In the Italian national survey, 11 of 14 pediatric robotic centers (78.6%) reported sharing the robotic platform with adult surgery [18, 24]. Finally, the literature identifies AI and algorithmic liability as an emerging area of medico-legal concern, although these issues remain conceptually distinct from conventional robotic platform malfunction. Overall, the reviewed literature supports a multidimensional approach to medico-legal assessment in robotic surgery, incorporating professional responsibility, institutional and organizational factors, device-related liability, and, prospectively, AI-related considerations. Importantly, the AI-related literature should not be interpreted as evidence of AI-related adverse events in the present cohort, in which all seven technical incidents were related to the robotic platform or operating environment.

## Discussion

There are only a few studies in the literature focused on medical liability in robotic assisted surgery, particularly within the field of pediatric RAS [8, 9, 14]. Our group previously addressed the medico-legal issues surrounding laparoscopic surgery some years ago [12, 13]. In conventional laparoscopy, the surgeon directly manipulates the instruments through the abdominal wall and therefore maintains a relatively direct mechanical relationship with the operative field. Although laparoscopic surgery is also dependent on complex technological equipment, liability in the event of an adverse outcome has traditionally been considered predominantly within the framework of professional conduct, surgical decision-making, and the appropriate use of the available equipment [12, 13].

Robotic surgery, however, reopens the debate with renewed complexity: the surgeon operates remotely from a console, and the instruments are mediated by a robotic interface rather than directly manipulated by hand [15–17]. This technological shift alters the traditional relationship between operator, device, and patient, introducing new medico‑legal considerations. This does not mean that robotic surgery automatically creates a different standard of care or transfers liability away from the surgeon. Rather, it expands the number of potential factors that may need to be considered when determining causation. A complication occurring during RAS should therefore not be attributed automatically either to operator error or to technological failure. The relevant question is whether the adverse event resulted from inappropriate clinical decision-making, incorrect use of the system, a technical malfunction, an organizational failure, or a combination of these factors.

As documented in our series of 205 robotic procedures [18], our group encountered technical platform-related issues in 7 cases (3.4%). Although these incidents did not result in intraoperative injuries or long-term clinical sequelae, they carried a high potential to cause catastrophic complications. For instance, any unintended instrument displacement during a sudden system shutdown or during critical latency intervals could irreversibly damage major blood vessels, bowel loops, or parenchymal organs. We clarified the nature of each incident by distinguishing between events directly attributable to the robotic platform and those related to the operative environment or human factors. Accidental power‑cable disconnections, for example, were categorized as environment‑related incidents. Importantly, the positioning of the robotic boom, column, and consoles depends on the operating room layout of the host department. Any irregular arrangement or cables left trailing on the floor may contribute - together with human factors - to accidental disconnections. These circumstances fall under the responsibility of the hospital facility and the host adult surgical department, which manages installation, calibration, and routine maintenance of the shared robotic platform. All incidents were documented according to internal hospital quality‑assurance protocols. Events involving components of the robotic system were communicated to the manufacturer through standard reporting channels. None of the incidents required manufacturer intervention during surgery, and no system shutdown persisted beyond immediate reconnection or reset. From a medico-legal perspective, contemporaneous documentation may be particularly relevant when the subsequent assessment of causation requires distinguishing a technical malfunction from an operator error, an environmental event, or an organizational failure.

Prompted by these findings, and in collaboration with an expert forensic physician, we sought to examine this paradigm in greater depth by benchmarking our institutional experience against the current international legal literature. Accordingly, two distinct forms of contemporary liability can be identified in this technological era: one arising from the integration of artificial intelligence (AI) and the other stemming from algorithmic and robotic medical technology. This paradigm shift raises unprecedented legal questions that challenge traditional medical malpractice frameworks:

- Who holds legal responsibility when a complication occurs during a robotic procedure?
- Is liability strictly limited to the console surgeon—as traditionally occurs in open or conventional laparoscopic surgery—or does it extend to the robot manufacturer, the healthcare institution, the software developer, or the biomedical supplier?

Furthermore, within the Italian legal framework, it is critical to evaluate how traditional concepts of negligence and surgical risk evolve beyond the statutory boundaries established by the Gelli-Bianco Law (Law 24/2017), particularly when strict product liability and algorithmic faults intersect with human conduct. In such scenarios, the burden of proof becomes a crucial judicial battleground, as courts must definitively establish whether a specific intraoperative human error was independently committed or explicitly induced by an underlying algorithmic or mechanical malfunction.

When examining these novel profiles of liability, a clear dichotomy emerges:

1. Liability associated with robotic infrastructure and device‑mediated surgical action: robotic surgery is a team‑mediated act involving the console surgeon, the bedside assistant, scrub and circulating nurses, and biomedical personnel [8, 19, 27–35]. Responsibility may therefore extend beyond the operator to the entire surgical team, particularly regarding system setup, intraoperative supervision, and adherence to safety protocols.
2. Liability arising from device malfunction or software‑related anomalies: although current pediatric robotic platforms (e.g., Da Vinci Xi) do not incorporate autonomous or self‑learning AI [25, 26], the literature highlights the growing relevance of algorithm‑based decision support systems and the medico‑legal implications of software‑mediated surgical actions [19–23]. In such contexts, distinguishing human error from device malfunction becomes a central judicial challenge.

This multi-factorial clinical environment yields three major medico-legal consequences:

- Shared (Multi-Subject) Liability: Accountability is no longer restricted to the console surgeon. It extends to the entire surgical team, including the table assistant (assistant surgeon), the scrub nurse, and the circulating nurse, insofar as system selection, intraoperative programming, and visual supervision become collective responsibilities [8, 22, 23, 33].
- Institutional and Organizational Liability: Healthcare institutions incur both contractual and tort (extracontractual) liability for organizational negligence. This includes the implementation of rigorous equipment maintenance protocols, formalized personnel training/credentialing [36–40], and the strategic selection of specific robotic devices [29–31, 40–43].
- Non-Delegable Duty of Supervision: Physicians cannot legally delegate their professional liability to an automated system. Surgeons remain fully responsible for understanding the technological limitations of the platform and must retain the competence to intervene promptly and transition to open or laparoscopic surgery in the event of a system malfunction.

This interplay between technical and organizational failure is particularly pronounced in pediatric surgery. As documented in national data, 78.6% of Italian pediatric robotic programs operate on platforms physically located within adult operating suites [24]. In these specific logistical models, the adult surgical departments hosting the platform retain primary administrative responsibility for the proper installation, calibration, and routine maintenance of the robot and its proprietary instrumentation [8, 33]. Consequently, when an adverse event occurs, errors may stem from institutional or organizational shortcomings rather than direct human malpractice. As a result, a new category of injury emerges, one that no longer derives exclusively from human error but may instead result from software defects, algorithmic bias, or machine malfunction [8, 22, 23, 33]. Accordingly, additional potentially liable parties enter the legal framework, including the robot manufacturer, software developer, and medical device supplier, alongside the physician and healthcare institution. In these circumstances, the legal provisions governing liability for defective products become applicable [8, 22, 23, 33]. To systematically address these issues, it is necessary to apply the doctrine of the “legal guarantor” within the context of civil and product liability. Under current European Union and national legal frameworks, three principal figures can be identified:

- The Manufacturer: Liable under strict product liability doctrines if a design, manufacturing, or software defect existed at the time the robotic platform was placed on the market.
- The Operator (or User): The professional clinician utilizing the system to derive a professional benefit. Notably, the European Union’s regulatory frameworks—including early legislative proposals—position the operator as a primary liable subject, especially concerning high-risk autonomous systems.
- The Owner or Holder: The healthcare institution or entity responsible for the physical control, environmental safety, and management of the robotic device.

In 2017, the European Parliament also proposed the introduction of mandatory insurance together with a compensation fund, recognizing that highly autonomous robotic systems make it increasingly difficult to identify a single legally responsible party. This evolving landscape is further defined by the European AI Act (2024), which strictly delineates responsibilities between the “provider” (the entity developing and marketing the AI/robotic system) and the “deployer” (the healthcare professional or hospital employing the system in clinical practice). Both stakeholders are legally mandated to implement proactive risk management measures. In the event of litigation, regulatory and judicial authorities will evaluate whether each party fulfilled their respective statutory obligations.

Amidst these technological and legal complexities, the procurement of informed consent remains a foundational and non-delegable milestone. Parents or legal guardians must be explicitly and comprehensively informed prior to surgery regarding the specific use of robotic technology. This disclosure must go beyond standard surgical risks to detail the unique technical risks associated with robotic systems, including potential mechanical failures, latency, or conversions to conventional surgery. The informed consent document must be signed by both the parents and the operating surgeon well in advance, preferably the day before the procedure. To strengthen the defensibility of this process against future litigation, it is highly advisable to have a neutral witness present during the clinical discussion where these technical risks and potential complications are explained. It represents a recommended best practice in several European centers, although it may vary across jurisdictions. Our 10-year institutional experience, combined with international literature, demonstrates that robotic surgery disrupts traditional liability frameworks. While liability in open or conventional laparoscopic surgery is predominantly attributed to the primary surgeon who physically manipulates the instruments at the bedside, RAS introduces a decentralized paradigm. Medico-legal responsibility in pediatric robotics cannot reside solely with the operator; it must be equitably shared among the surgeon, the healthcare institution, and the device manufacturer. Because adverse events may arise from a confluence of software defects, algorithmic anomalies, human actions, and organizational or maintenance shortcomings, developing explicit, multi-stakeholder shared accountability frameworks is imperative to ensure both patient safety and fair legal adjudication in modern surgical practice.

## Data Availability

No datasets were generated or analysed during the current study.
